# Variable Level of Dominance of Candidate Genes Controlling Drought Functional Traits in Maize Hybrids

**DOI:** 10.3389/fpls.2017.00940

**Published:** 2017-06-09

**Authors:** Ha Van Gioi, Mallana Gowdra Mallikarjuna, Mittal Shikha, Banduni Pooja, Shailendra K. Jha, Prasanta K. Dash, Arunkumar M. Basappa, Raveendra N. Gadag, Atmakuri Ramakrishna Rao, Thirunavukkarasu Nepolean

**Affiliations:** ^1^Division of Genetics, Indian Agricultural Research Institute (ICAR)New Delhi, India; ^2^Forage Crops Department, Maize Research InstituteHa Noi, Vietnam; ^3^National Research Centre on Plant Biotechnology (ICAR)New Delhi, India; ^4^Division of Seed Science and Technology, Indian Agricultural Research Institute (ICAR)New Delhi, India; ^5^Centre for Agricultural Bioinformatics, Indian Agricultural Statistics Research Institute (ICAR)New Delhi, India

**Keywords:** additive, adaptive traits, candidate genes, dominance, drought, functional traits, maize

## Abstract

Breeding maize for drought tolerance necessitates the knowledge on tolerant genotypes, molecular basis of drought tolerance mechanism, action, and expression pattern of genes. Studying the expression pattern and gene action of candidate genes during drought stress in the hybrids will help in choosing target genes for drought tolerance breeding. In the present investigation, a set of five hybrids and their seven parents with a variable level of tolerance to drought stress was selected to study the magnitude and the direction of 52 drought-responsive candidate genes distributed across various biological functions, *viz*., stomatal regulation, root development, detoxification, hormone signaling, photosynthesis, and sugar metabolism. The tolerant parents, HKI1105 and CML425, and their hybrid, ADWLH2, were physiologically active under drought stress, since vital parameters *viz*., chlorophyll, root length and relative water content, were on par with the respective well-watered control. All the genes were up-regulated in ADWLH2, many were down-regulated in HM8 and HM9, and most were down-regulated in PMH1 and PMH3 in the shoots and roots. The nature of the gene action was controlled by the parental combination rather than the parent *per se*. The differentially expressed genes in all five hybrids explained a mostly non-additive gene action over additivity, which was skewed toward any of the parental lines. Tissue-specific gene action was also noticed in many of the genes. The non-additive gene action is driven by genetic diversity, allele polymorphism, events during gene regulation, and small RNAs under the stress condition. Differential regulation and cross-talk of genes controlling various biological functions explained the basis of drought tolerance in subtropical maize hybrids. The nature of the gene action and the direction of the expression play crucial roles in designing introgression and hybrid breeding programmes to breed drought tolerant maize hybrids.

## Introduction

Maize (*Zea mays* L.) is one of the major crops in the world, with the highest genetic yield potential among cereals. Maize leads the world cereal production, with an annual production of 1,021 million tons from 183 million hectares and a global productivity of 5.5 tons per hectare. The crop has well established in global agriculture owing to its multi-faceted uses (Nepolean et al., 2013). Despite of high production and diverse utility, maize crop suffers from drought-induced yield losses in major portion of the world especially in tropical and sub-tropical regions. Further, changing climatic scenario is exaggerating the drought situation in maize growing areas of developing nations. Development of drought tolerant maize hybrids will be a viable option to cope-up with the changing climate and increasing water sacristy in agricultural production system. It is imperative to understand the mechanisms of drought tolerance and their molecular basis to develop drought-tolerant maize cultivars. Drought impairs various metabolic processes in maize, such as a reduction in chlorophyll content, weak transpiration and low photosynthetic activity. Several genes and proteins responding to stress were identified for various molecular and biological functions in different crops (Cushman and Bohnert, 2000; Sreenivasulu et al., 2004; Yamaguchi-Shinozaki and Shinozaki, 2005; Shikha et al., 2017; Thirunavukkarasu et al., 2017).

The expression of *AtMYB60* (Cominelli et al., 2005) and *AtMYB61* (Liang et al., 2005) in *Arabidopsis* facilitates stomatal opening and closure, whereas in rice, *SNAC1* (Hu et al., 2006) leads to stomatal closure. Up-regulated expression of *OsDREB2A* in rice increases the biomass in roots (Cui et al., 2011). The expression of NAC genes; *OsNAC10* and *OsNAC5* during desiccation causes enlarged roots and diameters, respectively (Jeong et al., 2010, 2013). In *Arabidopsis, AtMYB60* is known to promote root growth during the early stages of drought stress (Oh et al., 2011). In addition to alteration of root phenology, drought stress elevates the ROS production, which causes severe oxidative damage to the proteins, DNA and lipids. Plants have both enzymatic and non-enzymatic antioxidant scavenging mechanisms to cope up with stress by ROS (Mittler, 2002). Genes for ROS scavenging have already been isolated from major crops and model plants *viz*., maize, rice and *Arabidopsis*.

Photosynthesis is the main physiological event that is negatively affected by drought (Chaves and Chaves, 1991). Drought modulates photosynthetic metabolism through altering the expression of genes or proteins associated with drought tolerance (Lawlor and Cornic, 2002). In maize, *ZmPRK1* and *ZmMe3* code for *NADP malic enzyme 3*, whereas *ZmrbcL* codes for the large Rubisco subunit involved in photosynthetic activity during drought (Nguyen et al., 2009). The overexpression of some proteins, such as GolS1 and GolS2 in *Arabidopsis*, causes the accumulation of galactinol and raffinose in dry conditions (Taji et al., 2002; Nishizawa et al., 2008). Similarly, in Arabidopsis AtTPS1 increases the trehalose and trehalose-6-phosphate (T6P) for enhanced drought tolerance (Avonce, 2004).

Maize is a cross-pollinated crop, and the F1 hybrid from the heterotic parental combination is an output of a breeding programme. Thus, studying the gene expression in hybrids along with the parental lines is important to determine the gene action that will aid in the selection of genes as well as the parental lines for hybrid breeding programme. In 2003, Guo and his colleagues used a cDNA-AFLP approach to study the expression levels in maize endosperm tissue isolated from genotypes that display a range of heterosis for grain yield (Guo et al., 2003). A genome-wide gene expression analysis of two heterotic crosses revealed 7-9% differentially expressed genes in the shoots of rice seedlings from two sets of heterotic crosses (Zhang et al., 2008). A genome-wide transcriptome analysis in maize hybrids identified the existence of a positive association between the additive allelic expression of genes and a hybrid yield and heterosis. Furthermore, a negative correlation was revealed between the hybrid yield or heterosis and genes that exhibit a bias toward the expression level of the paternal parent (Guo et al., 2006). As per our literature survey there has been no-efforts on candidate gene based expression assay to understand the gene action and expression pattern in the parental inbred line and hybrids of subtropical germplasm. Hence, the present research is framed to understand the nature and direction of drought-responsive candidate genes expression in parents and the hybrids belonging to subtropical maize germplasm under drought stress and to understand the functional role of those candidate genes and to reveal the cross-talking of candidate genes in imparting drought stress tolerance.

## Materials and methods

### Plant material

A set of seven parents and five hybrids was selected for this experiment (Table 1). The parental lines include two drought-tolerant inbreds, HKI1105 and CML425, and five drought-sensitive inbreds, HKI1128, HKI161, LM13, LM14, and LM17. The hybrid panel includes one drought-tolerant hybrid ADWLH2 (HKI1105 × CML425), two moderately tolerant hybrids HM8 (HKI1105 × HKI161) and HM9 (HKI1105 × HKI1128), and two sensitive hybrids PMH1 (LM13 × LM14) and PMH3 (LM17 × LM14).

**Table 1:** Parental lines and their respective hybrids used in the experiment.

**S.No**	**Hybrid**	**Parents**	**Maturity group**	**Hybrid response to drought stress**
1	ADWLH2	HKI1105 × CML425	Medium	Tolerant
2	HM8	HKI1105 × HKI 161	Medium	Moderate
3	HM9	HKI1105 × HKI1128	Medium	Moderate
4	PMH1	LM13 × LM14	Late	Sensitive
5	PMH3	LM17 × LM14	Late	Sensitive

### Drought stress experiment

Seven parental inbreds and the five hybrids were grown under controlled glasshouse conditions at the National Phytotron Facility, ICAR-IARI, New Delhi. The glasshouse conditions include, 30/26°C (day/night) with relative humidity of 50–55%. All the genotypes were sown in plastic cups (6”) filled with sandy loam soil in a randomized complete block design and were replicated three times with three plants per replication (Thirunavukkarasu et al., 2017). Plants were watered at field capacity until the emergence of the third leaf. Stress treatment was given to each genotype after the emergence of the third leaf by withdrawing water and keeping them without water for five consecutive days. A second set of genotypes was maintained simultaneously with regular watering at field capacity to serve as a control (Min et al., 2016; Thirunavukkarasu et al., 2017).

### Candidate genes

A set of 52 drought-responsive candidate genes distributed across the maize genome was selected to understand their expression in the parental inbreds and the hybrids under a drought stress condition, of which, 14 genes were selected from a genome-wide association mapping study owing to their association with drought tolerant functions in maize (Thirunavukkarasu et al., 2013). The remaining 38 genes known for drought tolerance were selected from the public data base. In the preliminary search, several genes were collected from the Plant Transcription Factor Database (PlantTFDB) and MaizeGDB. The domains of the collected genes were searched using the Pfam database using a cut-off value of 1.0. The genes that satisfy the *E*-value of 1 were further used for analysis. The genomic sequences of all the genes were retrieved from Ensembl Plants (http://plants.ensembl.org/index.html). For each gene, the gene ontology, under different categories, such as molecular, cellular and biological functions, was identified using Blast2Go (Conesa and Götz, 2008). Then, the annotations of all the sequences were manually checked for drought-related functions. Finally, 38 genes with drought-related functions were short-listed and were further cross-checked by the Stress Responsive Transcription factor Database (STIFDB), which classifies the genes according to different stresses.

Selected genes were categorized into different drought-related functional groups (Table 2), including stomatal regulation (10 genes), root development (10 genes), ROS scavenging (10 genes), hormone signaling (12 genes), photosynthesis (5 genes), and sugar metabolism (5 genes). Features of genes, such as length and position in the genome, were identified using Phytozome (https://phytozome.jgi.doe.gov/pz/portal.html).

**Table 2:** Characteristics of the candidate genes under various functional categories selected for the gene expression assay.

**S.No**	**Gene ID**	**Ch**	**Gene**	**Function**
**STOMATAL REGULATION**				
1	GRMZM2G069365	4	*zhd 17*	ABA-dependent pathway
2	GRMZM2G071112	7	*zhd 13*	ABA-dependent pathway
3	GRMZM2G089619	2	*zhd 15*	ABA-dependent pathway
4	GRMZM2G122479	6	*me2*	Ion homeostasis-dependent pathway
5	GRMZM2G407181	1	*nced2*	ABA-dependent pathway
6	GRMZM5G858784	3	*nced3*	ABA-dependent pathway
7	GRMZM2G159724^*^	3	*me6*	Nucleotide binding, protein binding
8	GRMZM2G053384^*^	2	*PRC protein*	RNA binding
9	GRMZM2G102429^*^	2	*u-box*	Catalytic activity
10	GRMZM2G060465^*^	4	*ereb155*	DNA binding
**ROOT DEVELOPMENT**				
11	GRMZM2G015605	10	*nac1*	Auxin transport
12	GRMZM2G028648	6	*nac2*	Auxin transport
13	GRMZM2G090576	5	*nac3*	Auxin transport
14	GRMZM2G091819	10	*Flavin monoxygenase*	Auxin biosynthesis
15	GRMZM2G104400	8	*nactf38*	Auxin transport
16	GRMZM2G371345	10	*V-type PPase H+ pump*	Auxin transport
17	GRMZM2G003466	1	*ereb101*	Dessication tolerance
18	GRMZM2G124037	2	*dbf3*	Dessication tolerance
19	GRMZM2G432571^*^	5	*NBS-IRR partial*	Nucleotide binding
20	GRMZM2G134073^*^	8	*nac68*	DNA binding
**ROS SCAVENGING**				
21	GRMZM2G025992	7	*sod2*	Oxygen radical detoxification
22	GRMZM2G054559	3	*pld1*	Phospholipid hydrolysis
23	GRMZM2G066120	1	*mkkk11*	ROS homeostasis
24	GRMZM2G071021	3	*aldh3*	ROS homeostasis
25	GRMZM2G140667	2	*apx2*	ROS homeostasis
26	GRMZM2G172322	1	*gsr1*	H2O2 metabolism
27	GRMZM5G884600^*^	10	*GPx*	Catalytic activity
28	GRMZM2G059991	6	*sod3*	Oxygen radical detoxification
29	GRMZM5G822829^*^	10	*BHLH*	DNA binding
30	GRMZM2G367411^*^	5	*mkk6*	Kinase activity, nucleotide binding
**HORMONE SIGNALING**				
31	GRMZM2G056120	3	*artf11*	ABA-inducible stomatal closure
32	GRMZM2G057935	1	*phyC1*	Signaling network
33	GRMZM2G066867	5	*snrkII10*	ABA signaling network
34	GRMZM5G867568	3	*MAPKK3*	ABA signaling
35	GRMZM2G112240	4	*prh1*	ABA signaling network
36	GRMZM2G180555	9	*MKKK10*	Signaling network
37	GRMZM2G305066	8	*MKKK18*	Signaling network
38	GRMZM2G117851^*^	3	*bzip1*	Sequence-specific DNA binding
39	GRMZM2G083717^*^	1	*wrky14*	Sequence-specific DNA binding
40	GRMZM2G152661^*^	10	*camta5*	DNA binding, protein binding
41	GRMZM2G008250^*^	1	*NFY-A*	Sequence-specific DNA binding
42	GRMZM2G172327^*^	7	*myb14*	DNA binding, chromatin binding
**PHOTOSYNTHESIS**				
43	GRMZM2G012397	7	*psa6*	Photosystem I reaction center 6
44	GRMZM2G078409	2	*ploc2*	Electron transfer
45	GRMZM2G122337	6	*Ferredoxin 1*	Oxidation reduction process
46	GRMZM2G162200	4	*rca1*	Role in photosynthesis
47	GRMZM2G162282	4	*rca3*	Role in photosynthesis
**SUCROSE METABOLISM**				
48	GRMZM2G016890	10	*Sbe2A*	Starch biosynthesis
49	GRMZM2G058310	7	*amyb5*	Starch degradation
50	GRMZM2G130043	4	*ss5*	Hydrolysis of sucrose
51	GRMZM2G152908	9	*sus1*	Sucrose metabolism
52	GRMZM2G175423	1	*sodh1*	Cellulose hydrolysis

* Genes selected from GWAS experiment (Please refer materials and methods)

### Trait measurements

Root length (RL), chlorophyll content (CC) and relative water content (RWC) were measured in the stress and the control plants. Three plants per replication were used to measure the root length and other phenotypic traits. Utmost care was taken to avoid the damage to root system while removing the soil and measuring of root length. The RL was measured 5 days after the stress treatment in stressed and well-watered control seedlings and is expressed in centimeters. The CC (%) was recorded daily from day 1 to day 5 of the stress treatment period using a portable SPAD chlorophyll meter (SPAD-502, Minolta Camera Co. Ltd., Japan). The RWC was measured 5 days after the stress period using the following formula:

$$\begin{array}{l} \text{RWC }\left(\%\right)=\left[\frac{\text{FW-DW}}{\text{TW-DW}}\right]*100 \end{array}$$

where FW is the fresh weight, DW is the dry weight, and TW is the turgid weight (Barrs and Weatherley, 1962).

### Isolation of total RNA

After 5 days of stress treatment, the parent and hybrid seedlings were removed from the cup, and the shoot and root samples were collected. Total RNA from the shoots and roots was separately isolated using Qiagen RNeasy columns (Qiagen, Hilden, Germany). From the isolated RNA, 2.5 μg RNA was treated with DNase I (Promega, Madison, WI) as per manufacturer's protocol to remove any genomic DNA contamination. The concentration of the isolated total RNA was determined using a Thermo Scientific NanoDrop 1000 spectrophotometer (Thermo Scientific, Delaware, USA). One microgram of RNA was used to synthesize cDNA using an Affymetrix Kit (Santa Clara, California, USA).

### qRT-PCR

Primers for each gene were designed using IDT PrimerQuest (http://www.idtdna.com/scitools/applications/primerquest/default.aspx), and the 18s RNA coding gene was selected as the internal control (Nakashima et al., 2007). The primer pairs for the genes were designed to produce only a single desired amplicon in the quantitative real-time PCR (qRT-PCR). The details of the primer pairs of the genes are given in Supplementary Table S1.

One-step real-time qPCR was performed in an Mx3005P qPCR system (Stratagene, La Jolla, CA) using a SYBR Green-based PCR assay (with ROX as the optional reference dye). qPCR was performed using a total reaction volume of 25 μl, which consisted of 12.5 μl of the SYBR green RT-PCR master mix (Affymetrix, Santa Clara, California, USA), 1 μl of reverse transcriptase, 0.5 μl of ROX dye, 1 μl of cDNA, 2 μl of each primer, and 8 μl of nuclease free water. Reverse transcription was performed at 50°C for 30 min and was then terminated at 95°C for 10 min; PCR was then performed with 40 cycles of 94°C for 3 s, 58–60°C for 1 min, and 72°C for 30 s with three technical replicates.

The expression data for the genes in the different treatments, tissues, and genotypes were normalized by subtracting the mean reference gene CT value from the individual CT values of the corresponding target genes (ΔCT). The relative abundance of the transcripts was derived using the expression 2^−ΔΔCT^, where ΔΔCT is the difference between the ΔCT of the condition of stress treatment and the ΔCT of the control.

### Analysis of gene action

The quantitative measurement of the F_1_ hybrid expression level of each gene related to the average of the two parents (mid-parental level) was determined using a *d/a* ratio method (Guo et al., 2003, 2006). Considering *d* is dominant, *a* is additive, and μ is the mid-parental value (average of the parental expression), the dominant (*d*) is measured by the difference between the F_1_ (hybrid) and the average of the parents (μ), and the additive (*a*) is measured by the difference between the parent (either maternal or paternal) and the average of the parents (μ) i.e.,

$$\begin{array}{l} d=\text{F}1-\mu \text{ and a}=\text{Parent}-\mu \end{array}$$

In case of a complete dominant gene action of the P_1_ (maternal) allele, F_1_ = P_1_, then *d/a* = 1. Similarly, *d/a* = −1 explains the complete dominant gene action of the P_2_ (paternal) allele. In case of an additive gene action, if F_1_ = μ, then *d/a* = 0.

Based on the above-mentioned concept, the transcript expression level is considered a phenotype of each gene, and the F_1_ hybrid expression was measured relative to the parental expression (Guo et al., 2003). In the case of multiple loci, “*a”* and “*d”* represent the composite effect of the respective gene action. Genetically, the genome of an F_1_ hybrid constitutes one dose of a genome from each parent, and an additive allelic expression in the hybrid would be expected to be equal to the mid-parental value [(maternal + paternal)/2].

From the actual expression data of the parents and the respective hybrids, the deviation of the F_1_ hybrid from the average of the two parents was calculated as *d* = F_1_ – μ. Then, the deviation of the maternal parent from the average was calculated as *a* = Parent _maternal_ – μ. The *d/a* ratio was then used to measure the gene action in the hybrids. If *d/a* = 0, this indicates additive gene action; if *d/a* = 1, this indicates the dominant gene action where the hybrid expression is skewed toward a maternal parent; if *d/a* = −1, this indicates a dominant gene action where the hybrid expression is skewed toward the paternal parent; if *d/a* > 1, this indicates an over-dominant gene action where the hybrid expression is skewed toward the maternal parent; and if *d/a* > − 1, this indicates over-dominant gene action where the hybrid expression is skewed toward the paternal parent, considering that the maternal parental expression is higher than the paternal parent expression. When the paternal parent expression is higher than the maternal parental expression, then the sign of the *d/a* ratio is reversed in over-dominant situations.

If the *d/a* ratio is between −0.5 and +0.5, this is considered an additive gene action, where both parental lines contribute equally to the gene expression, or the F_1_ hybrid expression is equal to that of the parental mean. If the *d/a* ratio > ±0.5, this is considered a non-additive gene action that is skewed toward any one of the parental lines.

## Results

### Response of the parental lines and hybrids to drought stress

**Figure 1:**
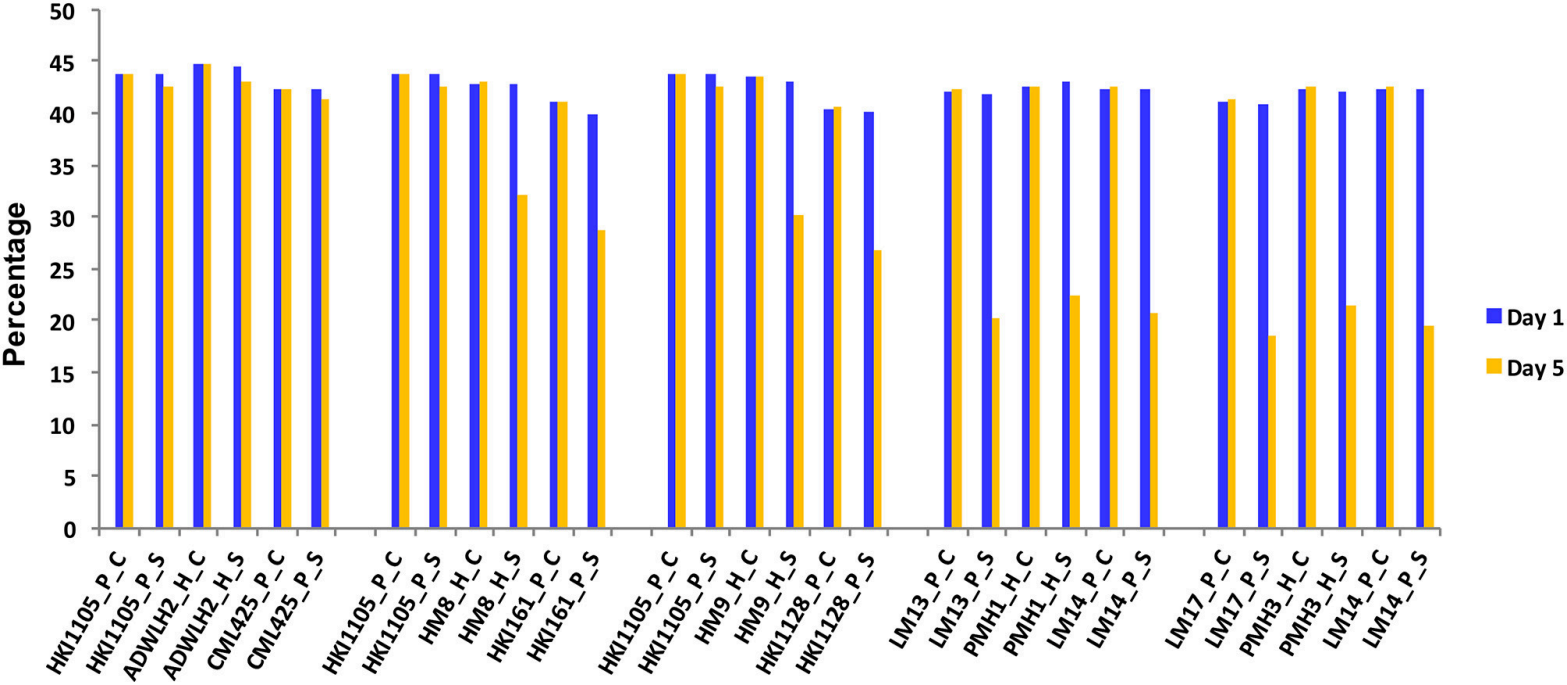
Change in the chlorophyll content in the parents and the respective hybrids under drought stress. Day 1 and Day 5 are the first and last day of the stress experiment, respectively. P, H, C, and S stand for parent, hybrid, control, and stress, respectively.

**Figure 2:**
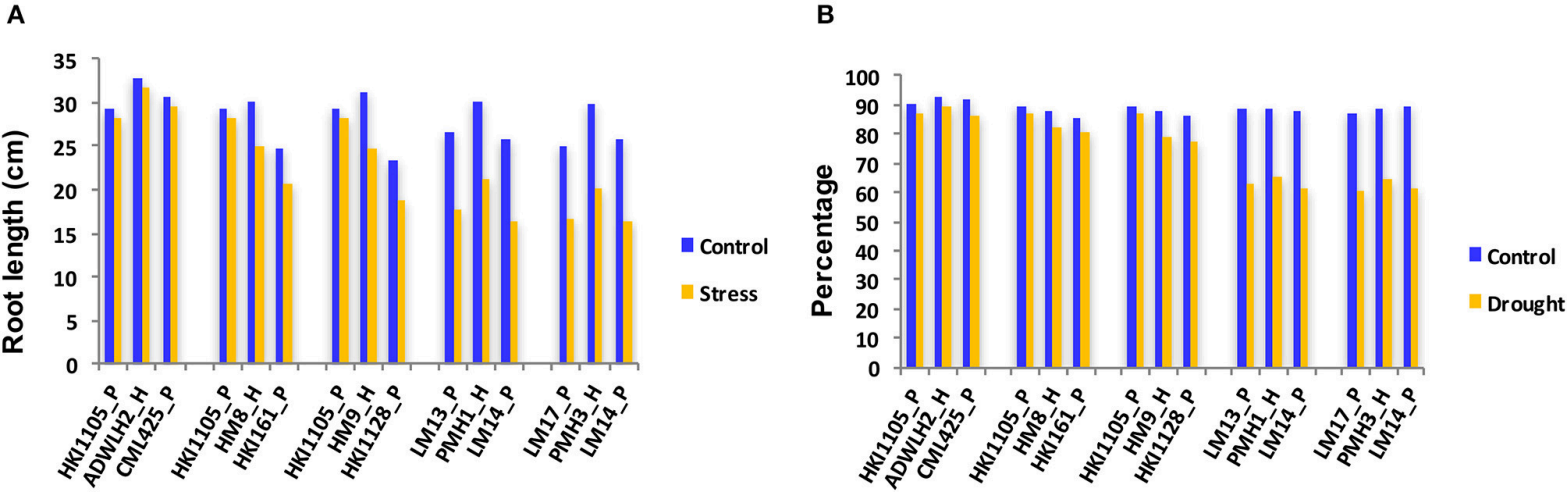
Root length **(A)** and the relative water content **(B)** of the parental lines and their respective hybrids under drought stress and control conditions on Day 5 of the stress. P and H stand for the parent and hybrid, respectively.

The reduction in the CC was a mere 2.5% in the parents HKI1105 and CML425 and was 3.2% in ADWLH2 on day 5 of stress compared to day 1 (Figure 1). The parents HKI161 and HKI1128 showed an 11.1 and 13.5% reduction, respectively, in the CC on day 5 compared with day 1 under the stress condition. Hybrids HM8 and HM9 showed more reduction in the CC to the level of 10.6 and 12.9%, respectively, on day 5 of the stress. A severe reduction in the CC (~50%) was noticed in the sensitive parents LM13, LM14, and LM17 and their hybrids PMH1 and PMH3 under the stress condition compared to their respective WW control. The growth of the roots under the stress condition in HKI1105 and CML425 and their hybrids was comparable to their respective WW control on day 5 since the reduction in the RL was ~2% (Figure 2A). An approximately 10% reduction in the RL was observed under stress in parents HKI161 and HKI1128 and in their respective hybrids HM8 and HM9 compared with the respective WW control. The RL was reduced more than 21% in HM8 and HM9 compared to ADWLH2 under the stress condition. LM14 showed a greater reduction in root growth (36%), followed by LM13 (34.1%) and LM17 (33.3%) compared with the respective WW control. The RWC, on day 5, was reduced to 3.5% in HKI1105, 6.2% in CML425 and 4% in ADWLH2 under the stress condition, and the RWC was further reduced in parents HKI161 and HKI1128 to the levels of 5.6 and 10.5%, respectively, under the stress condition compared to their respective WW control (Figure 2B). An approximately 30% reduction in the RWC was observed in all three of the sensitive parents (LM13, LM 14, and LM 17) as well their hybrids (PMH1 and PMH3).

### Gene expression pattern and gene action

Five hybrids and their respective parental lines were exposed to stress, and the expression level of 52 candidate genes was measured under the stress condition. These 52 genes represent six important functional categories, including stomatal regulation, root development, ROS scavenging, hormone signaling, photosynthesis, and sugar metabolism.

#### Stomatal regulation

**Figure 3:**
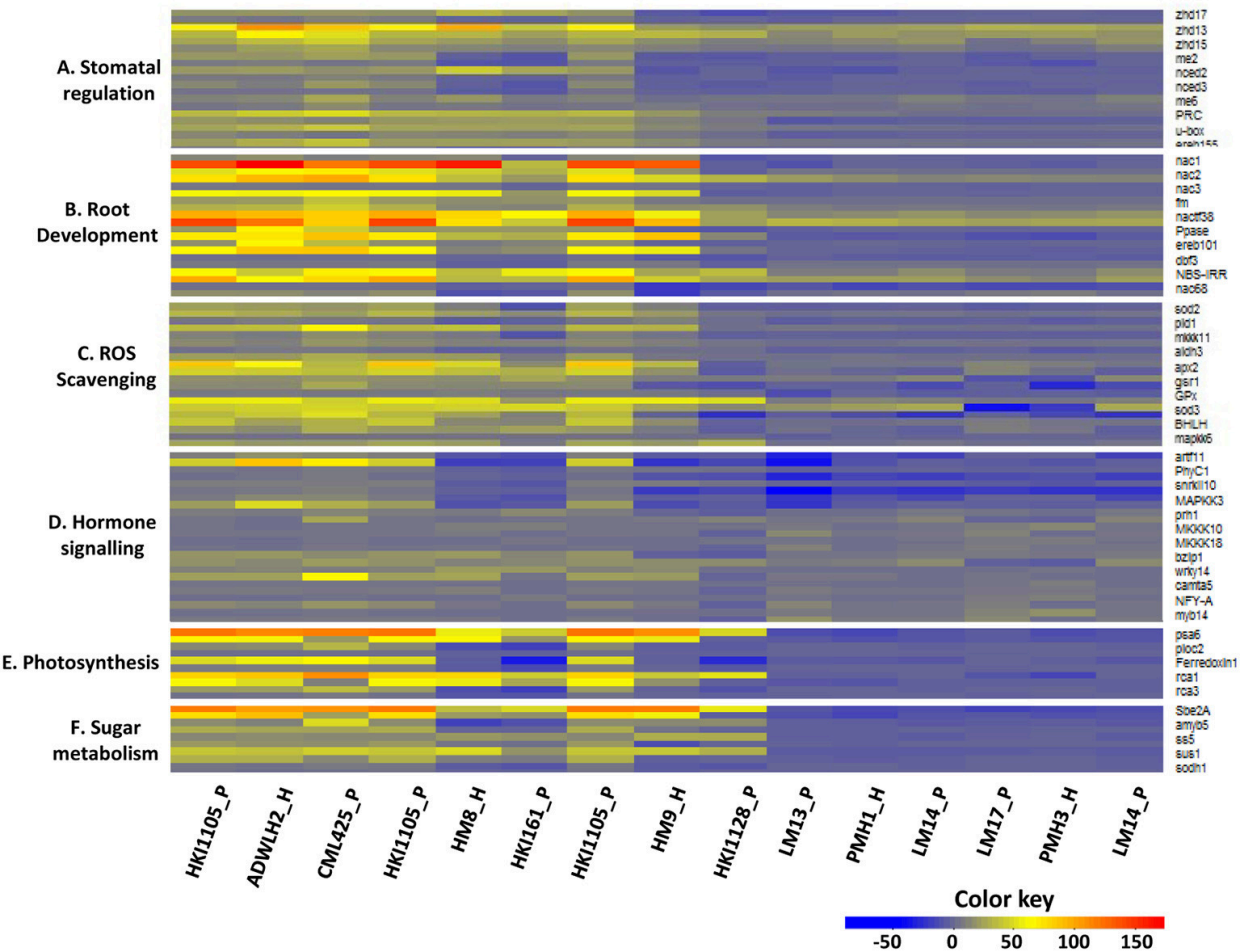
Differential expression pattern of the candidate genes in the shoot and root tissues of the parents and the respective hybrids. P and H stand for the parent and hybrid, respectively. The upper part of the heat map bar for each gene represents the shoot, and the lower part represents the root. **(A)** Stomatal regulation, **(B)** Root development, **(C)** ROS Scavenging, **(D)** Hormone signaling, **(E)** Photosynthesis, and **(F)** Sugar metabolism.

The expressions of all 10 genes in HKI1105 and CML 425 and in their hybrid (ADWLH2) were up-regulated both in the root and shoot tissues but to a greater extent in the shoots (Figure 3A). *u–box* protein and *ereb155* in the maternal parent HKI1105 were expressed three times than in the paternal parent CML 425 in the roots. Many genes in HM8 and HM9 and in their parents (HKI161 and HKI1128, respectively) were positively up-regulated, and the level of expression was lower than that of the tolerant lines HKI1105 and CML425. In all the negatively expressed gene scenarios of the LM13 and LM17 parental combination, the maternal parent LM13 showed a higher level of down regulation than the maternal parent LM17, whereas in the LM17 and LM14 parental combination, neither of the parents showed a higher level of negative expression over the other parent.

**Figure 4:**
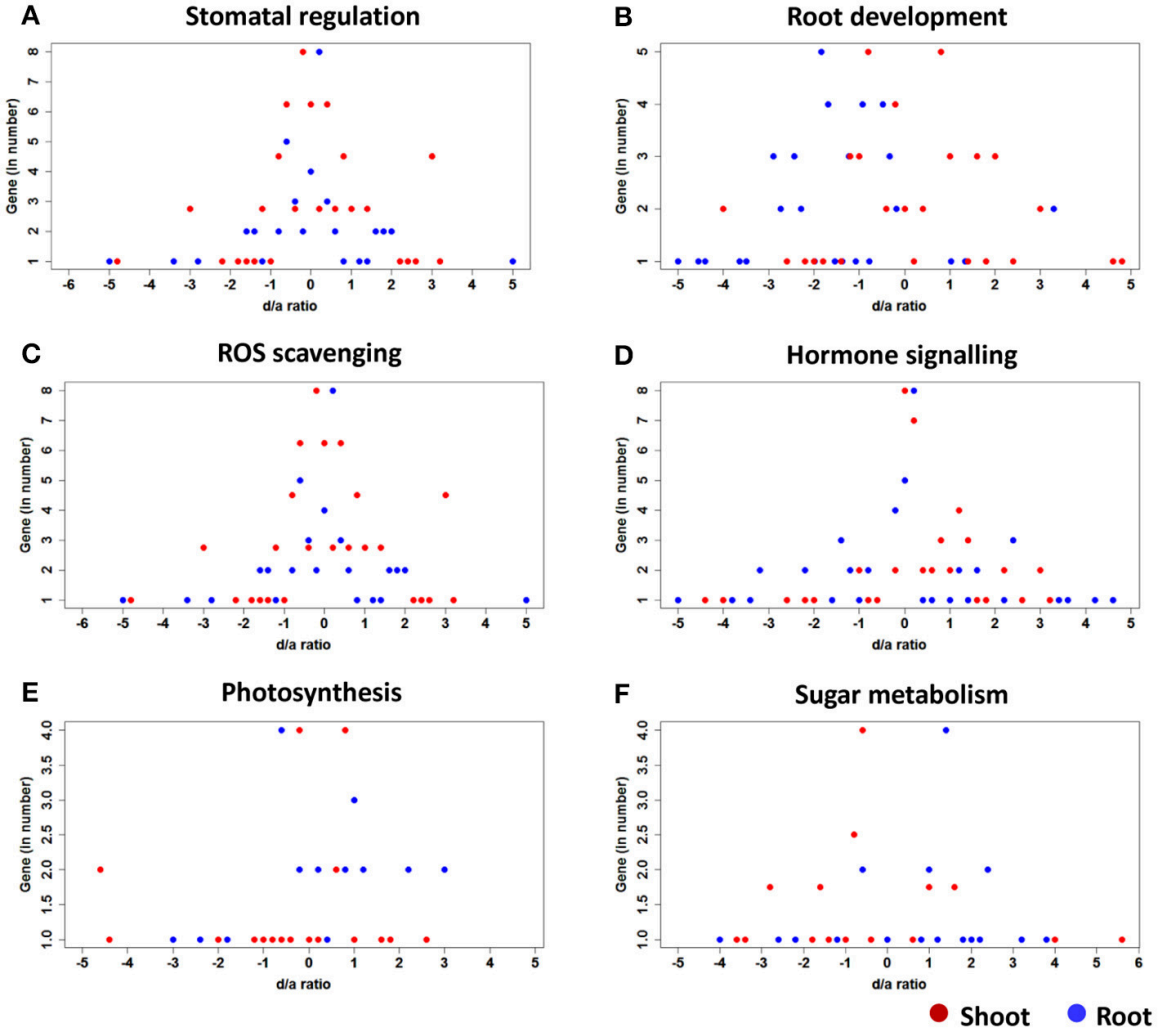
Patterns of the gene action of the candidate genes controlling various functional traits of the hybrids under stress. **(A)** Stomatal regulation, **(B)** Root development, **(C)** ROS scavenging, **(D)** Hormone signaling, **(E)** Photosynthesis, and **(F)** sugar metabolism.

Four of the 10 genes (*zhd 15, PRC protein, u–box*, and *ereb155*) showed additive gene action except in one case where *zhd15*, in the roots of PMH3, showed a slightly higher activity toward the paternal parent (Figure 4A). The remaining six genes expressed either dominant or over-dominant gene action in all five of the hybrids. However, the degree and the direction of dominance differed among the genes and the hybrids. *me6* in the shoots of HM8 showed a maximum degree of dominance of 5.58 by the maternal parent HKI1105, followed by 4.55 for PMH3 by the paternal parent LM17. All ten genes in all five hybrids showed skewed expression toward the maternal parents in both the tissues except in the shoots of HM9 for *me6*.

#### Root development

All the selected genes for studying root development were highly up-regulated in the roots and shoots of ADWLH2 and its parents but to a greater extent in the roots. *nac1* and *nac3* were expressed more than 10 times in the roots as compared to shoots in the inbreds HKI1105 and CML425 and in corresponding hybrid ADWLH2, which explained the importance of these genes under the stress condition for drought tolerance (Figure 3B). Two genes (*ereb101* and *nac68*) were negatively expressed in the paternal parent HKI161 and reduced the transcript level in its hybrid HM8. Of the seven genes that were down-regulated in the shoots of HKI1128, four were reduced at the transcript level in the hybrid HM9. The paternal parents LM13 and LM17 of PMH1 and PMH3, respectively, expressed higher levels of down-regulation in both the tissues for *nac1* and *nac3* and *V–type PPase H*+ *pump* over the common paternal parent LM14. Although the rest of the genes in both the tissues were up-regulated, the level of up-regulation was several folds less when compared to the tolerant lines HKI1105 and CML425.

The expression of two genes (*nac2* and *nactf38*) in all five hybrids in both shoots and roots were close to the midpoint level, which explained the additive nature of those genes, but PMH3 showed a dominant gene action in the shoots for *nac2*. The remaining eight genes deviated from the midpoint at varying levels in all the five hybrids (Figure 4B). *NBS-IRR partial* in the shoots of AWDLH2, *flavin monooxygenase* in the roots of PMH3 and *nac3* in the shoots of ADWLH2 showed a higher level dominant deviation toward the maternal parent (7.96, 6.78, and 5.42, respectively). All the hybrids expect for PMH1, showed maternal influence for the *NBS–IRR–partial* expression in both the shoots and roots. Three hybrids (HM8 and HM9 and PMH1) showed all paternal influence for *nac68*, whereas the expression of this gene in the rest of the hybrids was largely contributed by the maternal parents.

#### ROS scavenging

All ten genes tested for ROS scavenging activity showed a high level of up-regulation in ADWLH2, and its parents were probably one reason for the tolerant behavior of the parents and the hybrid. Four genes (*pld1, aldh3, GPx*, and *mkk6*) showed root-specific expression in the parental lines of ADWLH2 since, the level of expression was 4 to 10 times higher than that of the shoot tissue under the drought condition. Most of the up-regulated genes in HKI161 and HKI1128 were less expressed compared to the tolerant maternal parent HKI1105. The down-regulation of two genes, *pld1* and *aldh3*, in the shoots of HKI161 reduced the transcripts level in its hybrid HM8. The expression level of ROS scavenging genes in the parental lines PMH1 and PMH3 were very low in both the shoot and root tissues. *sod2, pld1, mkkk11, aldh3, GPx*, and *mkk6* showed shoot-specific down-regulation in PMH1 and PMH3 and in their parents (Figure 3C).

Three genes (*sod2, apx2*, and *sod3*) showed additive gene action in all the hybrids. However, the gene action of one of the genes, *apx2*, in the shoots and roots of PMH3 was not uniform. *Phospholipase D* (*pld1*) in the shoots of HM9 explained the highest level dominant deviation (4.58) toward the maternal parent, followed by *GPx* in the shoots of HM8 (4.16). Several genes showed over-dominance in the tissues of one or another hybrid, but *mkk6* showed over-dominance in the seedlings of all the hybrids except in the roots of HM8. *ereb101*, in all hybrids except in the shoots and roots of PMH1, and *mkkk11*, in all hybrids except in the roots of HM8 and the shoots of PMH3, showed maternal deviation in both root and shoot. The remaining genes did not show any clear-cut parental preference in the tissues of all hybrids (Figure 4C).

#### Hormone signaling

All 12 genes were positively regulated in the seedlings HKI1105 and CML425 and in their hybrid ADWLH2, with the highest level of expression in *artf11*. *artf11, phyC1, snrkII10*, and *MAPKK3* were the four genes that showed complete down-regulation in both tissues of the paternal parent HKI161 as well as in its hybrid HM8. Six root-specific negative expressions in HKI1128 (*MKKK10, MKKK18, wrky14, camta5, NFY–A*, and *myb14*) reduced the level of expression in the hybrid HM9, and this could be the reason for the moderate level of tolerance of the hybrid HM9 over ADWLH2 despite of having a common tolerant maternal parent HKI1105 (Figure 3D). More genes were down-regulated in the late maturing hybrids PMH1 and PMH3 and their parental lines over the medium maturing lines. *artf11, phyC1, snrkII10*, and *MAPKK3* were the common genes showing down-regulation in both the tissues of the hybrids PMH1 and PMH3 and their respective parents.

All the hormone signaling genes, in fact, followed dominant gene action and skewed toward one of the parental lines. The *d/a* ratio of *MKKK18* in the tissues of all the hybrids was more than 1 (except in the roots of HM9), which indicated the over-dominant action of the gene. *phyC1* in the shoots and *MAPKK3* in the roots of the hybrid of ADWLH2 showed the highest degree of dominance toward maternal and paternal parents (5.26 and −5.68, respectively). *artf11* and *MAPKK3* showed maternal dominance in all the hybrids except in PMH3 where it was paternal. However, in other cases, the degree and the direction of dominance were distributed across the hybrids and the tissues (Figure 4D). HM8 expressed paternal dominance for *artf11, phyC1, snrkII10*, and *MAPKK3* in the shoots and roots, and *artf11, phyC1, MAPKK3*, and *bZIP1* showed paternal dominance in HM9. Of the 12 genes studied, eight genes skewed toward the paternal parent LM14 in the hybrid PMH1 in both the tissues.

#### Photosynthesis

All five genes showed a clear-cut shoot-specific up-regulation in the hybrid ADWLH2 and its parents, with a higher level of fold change for *psa6* (>114). Three genes (*ploc2, Ferredoxin 1, rca3*) were negatively expressed in the parental inbreds HKI161 and HKI1128 and hybrids HM8 and HM9. Two root-specific down-regulations (*psa6* and *rca1*) in the paternal parent HKI1128 were observed but did not affect the positive expression of its hybrid HM9 owing to effect of maternal parent HKI1105. All the photosynthetic genes were down-regulated in the hybrids PMH1 and PMH3 as well as their parental lines (Figure 3E). *psa6* showed the highest negative expression in the shoots and roots compared to other genes in both the hybrids.

*Ferredoxin 1* in the shoots and roots of all the hybrids and *rca3* in the roots of PMH3 explained the additive gene action. The maternal influence in PMH3 was so strong for *rca1* since the *d/a* ratios in the shoots and roots were 2.85 and 2.5, respectively. *ploc2* shared the highest degree of paternal dominance in the root tissue of PMH1 and PMH3. *psa6* showed a significant level of maternal over-dominance in the shoots (*d/a* = 2.85), whereas the roots showed a higher level of paternal dominance (*d/a* = −5.46) in the hybrid PMH1 (Figure 4E). In all four of the non-additive genes of ADWLH2, the expression in the tissues was completely influenced by the HKI1105 over CML425 except in the shoots for *psa6*. In HM8, two genes (*ploc2* and *rca3*) were skewed toward the paternal parent HKI161, and in HM9, three genes (*ploc2, rca1*, and *rca3*) were dominated by the paternal parent HKI1128.

#### Sugar metabolism

Three genes (*Sbe2A, sus1*, and *sodh1*) showed shoot-specific positive expression in HKI1105, CML425, and the hybrid ADWLH2. The hybrid HM8 and its sensitive paternal parent HKI161 showed a negative regulation in the shoots compared with the roots for *amyb5* and *sodh1*. Four genes (*Sbe2A, amyb5, ss5*, and *sus1*) expressed a root-specific down-regulation in HKI1128, of which, two (*ss5* and *sus1*) were also reduced at the transcript level in the root tissues of its hybrid HM9. All the genes were down regulated in both root and shoot tissues of PMH1 and its parents, and the magnitude of the down-regulation was higher in *amyb5* as compared to other genes of sugar metabolism. PMH3 also showed a total down-regulation for all genes, and except for *sodh1*, the other genes showed a shoot-specific down-regulation in the hybrid as well as in both parents (Figure 3F).

All five genes showed a dominant gene action similar to that of hormone signaling genes (Figure 4F). The degree of maternal dominance reached a maximum of 5.5 in the roots of the PMH1 hybrid for *Sbe2A*, followed by the roots (3.84) of the same hybrid for *ss5*. In contrast, a significant dominant deviation toward the paternal parent was observed in the shoots of ADWLH2 (−4.35), followed by in the roots of PMH3 for *ss5*. Out of 50 possible cases across the five genes, two tissues, and five hybrids, 28 cases showed paternal dominance for sugar metabolism. *Invertase 1* (*ss5*) showed a dominant deviation toward the paternal parent CML425, whereas *sus1* showed a deviation toward the maternal parent HKI1105, and the rest of the genes skewed toward either of the parents.

## Discussion

### Response to stress

The CC, RL and RWC of the drought tolerant parents (HKI1105 and CML425) were on par or slightly lower under stress than their WW controls. The performance of the hybrid ADWLH2 was also better under stress since its parents (HKI1105 and CML425) were highly tolerant to drought stress. There was a gradual reduction in all three parameters in the parents HKI161 and HKI1128. Despite the sensitive paternal parents, the growth performance of the hybrids HM8 and HM9 was moderate under stress since the maternal parent (HKI1105) in both hybrids was stress tolerant.

A robust root system is very important to absorb water, to maintain a higher level of water potential in the aerial systems and for the ability to transpire for a longer duration under stress conditions (Hammer et al., 2009). The presence of a good root system in the tolerant lines provided an advantage for their growth and development under the water stress condition. In water-deficient soils, a tolerant plant adapts and modifies its root architecture in a variety of ways (Herder et al., 2010). Leaves with a good amount of water and nutrient content are essential to support the root system (Price et al., 2002). The high RWC with a high CC in the tolerant lines under the stress conditions maintained the water potential as well as provided an adequate level of photosynthesis and nutrients to the plant system.

### Allelic contribution

**Figure 5:**
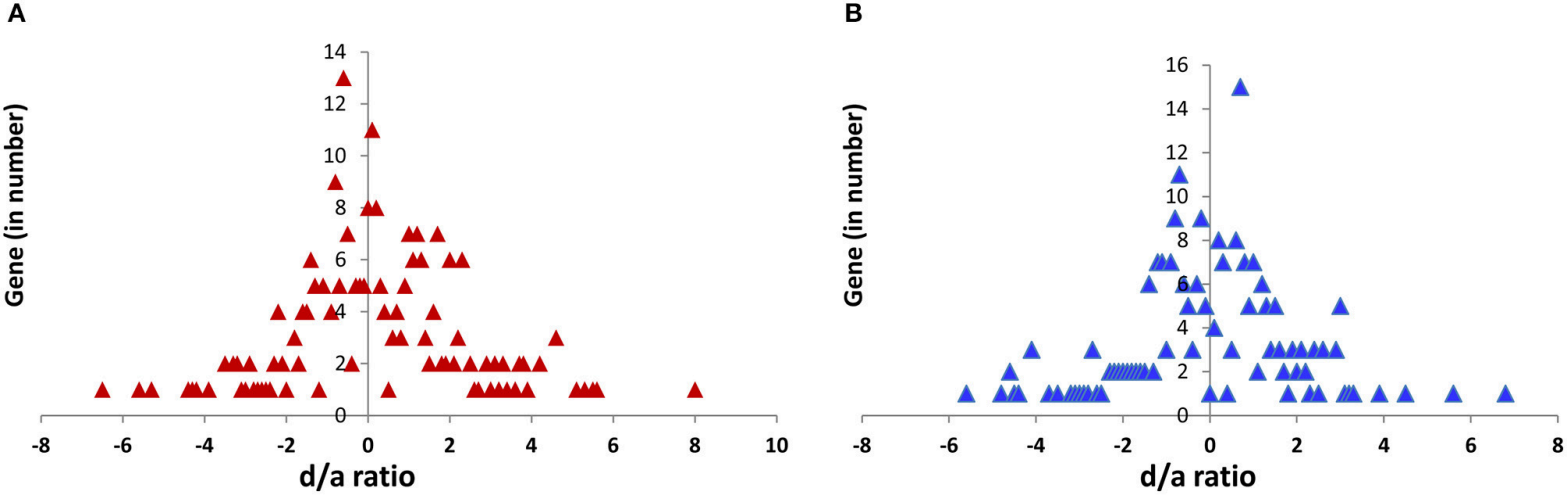
Global pattern of the d/a ratio for the 52 candidate genes under various functions in the shoot **(A)** and root **(B)** tissue of five hybrids.

The *d/a* ratio indicated that the expression of genes in the shoot and root tissues of the hybrids followed three patterns, including a mid-parental level (additive), one that was similar to that of the parental line (dominant) and one that fell outside of the parental expression (over-dominant) (Figure 5). The *d/a* ratio also showed a more or less equal distribution of dominance from both of the parents in the hybrids; however, the maternal influence was slightly higher (216) over the paternal (207) for all the genes, hybrids and tissues combinations.

In this experiment, the direction of influence by the parental lines is of great significance in all five hybrids. The direction of skewness did not affect the ADWLH2 performance under stress since the alleles from both parents contributed to the drought tolerance. However, the direction is very important for the hybrids HM8 and HM9, since one of the parents, HKI1105, was stress tolerant and the paternal parents, HKI161 and HKI1128, respectively, were not tolerant. Nearly 40% of the genes in HM8 and HM9 in the different functional categories showed dominance or over-dominance toward their respective sensitive parent HKI161 and HKI1128 in the shoots and roots. The influence of the sensitive parents in the hybrids reduced the transcript level in the respective hybrids, thereby affecting the crucial biological function under the stress condition. However, other genes showed either an additivity or non-additivity toward the tolerant parent HKI1105, which reduced the level of damage caused by stress and provided the necessary support for maintaining biological activities. PMH1 and PMH3 were sensitive to stress, and since their respective parents were stress sensitive, the transcript level in the hybrids also either followed the mid-parental level or was dominant toward the parents.

The gene action does not depend upon the parent *per se*, whereas it is characterized by the combination of the parental lines involved. For example, *me2* and *nced2* in the stomatal regulation category in AWDHL2 showed dominance toward the parent HKI1105 in the shoots and roots, whereas the genes in HM8 and HM9 showed dominance toward the other parent, HKI161 and HKI1128. A hormone signaling gene, *camta5*, explained the over-dominance toward LM14 in PMH1, but the same gene showed over-dominance toward the parent LM17 in PMH3. The variation in the direction and the magnitude of the same genes in the different hybrids, especially in the hybrids involving one common parent, suggested that the extent of the genetic diversity between the parents also decides the gene action (Stupar et al., 2008).

The gene action for a given gene may either be dominant toward any one of the parents or additive in nature. On a very rare occasion, the gene action was switched from dominant to additive for any one of the tissues. In addition, the genes that showed non-additive gene action were tissue-specific. For example, *aldh3* showed non-additive gene action toward CML425 in the shoots, and the same gene skewed toward the maternal parent HKI1105 in the roots. The tissue-specific expression of genes is one of the factors influencing the nature of gene action (Guo et al., 2003, 2004). A variation in the gene sequence or an allelic polymorphism will produce differential levels of transcripts, which is a major reason for the presence of variations in the transcript level. The structural variations and polymorphisms at the nucleotide level are one of the reasons for the transcriptional variation of the candidate genes under stress conditions (Messing and Dooner, 2006; Stupar and Springer, 2006). The intra-specific variation in the transcripts is attributed to the *cis*-acting factors in the genome (Stupar and Springer, 2006; Springer and Stupar, 2007).

Nearly 80% of the drought-responsive candidate genes explained partial-, complete-, or over-dominance toward any one of the parents. Several studies in *Drosophila* (Gibson et al., 2004), *Arabidopsis* (Vuylsteke et al., 2005), and maize (Stupar et al., 2008) have found that the expression of genes falls outside of the parental range. The non-additive action outside the parental range in the hybrids could be due to novel gene regulation owing to complementation of alleles under stress conditions (Stupar et al., 2008). The genome-wide expression of genes in hybrids was predominantly additive in nature. However, a non-additive gene action from a few to several loci in the genome was observed in various crops (Auger et al., 2005; Guo et al., 2006; Swanson-Wagner et al., 2006; Uzarowska et al., 2007; Pea et al., 2008; Li et al., 2009).

A variation in non-additive gene action might be due to the tissue-specific expression of genes (shoots or roots), and the specific growth conditions in which the genotypes were exposed (seedling or flowering and control or stressed). A non-additive gene action, especially over-dominance of the crucial genes under the stress condition, is also the result of the linked-loci of those genes. Since, drought tolerance is a complex trait, and various component traits are involved in stress tolerance, these traits are supported by a cascade of pathways inter-linking with each other. Further, these pathways are triggered in response to stress and results in the abundance of specific transcripts that are needed for the survival of the plant and to maintain various biological functions. Linked loci also play a critical role in the overexpression of genes (Swanson-Wagner et al., 2006) under the stress condition. The over-dominant gene action of these genes under the stress condition was also contributed by a range of post-transcriptional processes, including splicing, translation, protein folding and stabilization. Additionally, the role of small RNAs, such as microRNA and small interfering RNA (siRNA) cannot be ignored (Swanson-Wagner et al., 2006).

### Role of candidate genes in drought tolerance

Drought stress in plants triggered a series of stimuli and activated several genes to act in response to stress. The series of genes involved in the stress mechanism are the signaling genes that activate other down-stream genes, i.e., genes that protect the cellular components and functions (uptake of water, ions and nutrients, regulation of photosynthesis, and etc). Many of the genes that were part of the signaling cascade were activated in the tolerant lines under stress, which explained that these genes were actively involved in sensing the stress and were able to trigger other genes. One of the key hormones is ABA, which plays a variety of roles under stress conditions, including stomatal regulation. An increased accumulation of ABA during stress stimulates the closure of stomata and decreases the transpiration rate under stress conditions (Boursiac et al., 2013).

The ABA signaling pathway comprises three components, which are a group of ABA receptors, including *protein phosphatase 2C* and *SnRK2*. *SnRKs* are serine/threonine protein kinases that represent key regulators of plant responses to different stresses (Soon et al., 2012). *SnRK2* is an important signaling molecule that phosphorylates its downstream targets, including the transcription factors NAC, bZIP, HSF, MYB, WRKY, and RAV1 (belonging to the AP2–ERF family) (Furihata et al., 2006; Fujita et al., 2009; Kim et al., 2012; Feng et al., 2014). ERF is another drought-responsive transcription factor that is stimulated under the effect of ABA but is integrated with other two hormones, jasmonic acid and ethylene, which induce the closing of stomata (Cheng et al., 2013).

Root development is strongly influenced by abiotic stress conditions. A subfamily of NAC transcription factors is involved in various abiotic stresses such as drought, cold, salinity (Mao et al., 2012). Under a water stress condition, vacuolar proton pumps reduce the water potential. The *V–type PPase pump* enhances ion homeostasis, which regulates the osmotic balance and, hence, copes with drought (Li et al., 2008; Pasapula et al., 2011). Oxidative stress commonly occurs when the plant undergoes any kind of stress; the production of antioxidants is one of the ways the plant copes with the stress. In stress conditions, *ascorbate peroxidase* functions as a H_2_O_2_ reductant in ROS detoxification (Foyer and Halliwell, 1977). Under oxidative stress conditions, *ascorbate peroxidase* reduces H_2_O_2_ to water and mono-dehydroascorbate. Under drought stress, *glutathione reductase* activity is increased in the roots and leaves, which might facilitate the defense against ROS (Gallé et al., 2013). Additionally, *SOD* and *glutathione peroxidase* are also involved in ROS scavenging (Dietz et al., 2006). Recent advances have also emphasized the role of *MAPK* under drought stress in ROS detoxification (Ning et al., 2010).

In response to drought stress, photosynthetic activity is reduced because of a decline in stomatal conductance as well as Rubisco activities, resulting in lower carbon fixation followed by an over reduction in the components of ETS system. The up-regulation of the *Rubisco activase* precursor under the drought stress condition reveals that a higher activation state could also have affected the Rubisco activity (Ramachandra Reddy et al., 2004). Another transcription factor, *CAMTA*, is identified as a regulator of the photosynthetic machinery where the T-DNA insertion line of *AtCAMTAs* is observed at a low photosystem II efficiency under drought stress (Pandey et al., 2013). Abiotic stresses lead to major alterations in carbohydrate metabolism and most likely modulate metabolism sugar signaling pathways, which interact with stress pathways. Drought stress affects sucrose metabolism and decreases the activities of soluble and insoluble forms of *invertases* (Zinselmeier et al., 1995, 1999). *Sucrose synthase* enzyme is a key enzyme involved in the synthesis and cleavage of sucrose. Under water deficit conditions, the activity of *sucrose synthase* is enhanced in the cleavage direction and increases the sucrose synthesis (Castrillo, 1992; Yang et al., 2000).

## Conclusions

The tolerant genotypes HKI1105 and CML425 and their hybrid ADWLH2 performed better under drought stress due to the activation of various genes controlling different molecular functions. The genes played individual as well as cumulative roles in maintaining crucial biological functions under the stress condition. The cross-talk of these pathways also played vital role in controlling a cascade of molecular activities under stress conditions. The absence of such gene regulation in the sensitive inbreds and the hybrids is the reason for the susceptibility under drought stress. The role of these candidate genes is very important for understanding the drought tolerance in hybrid genotypes and designing strategies to breed drought-tolerant maize genotypes.

The gene action of the differentially expressed genes was either additive or non-additive across all the hybrids and tissues. The non-additive cases switched to any of the parents, depending upon the parental combination and showed tissue specificity in some of the hybrids. The genes mostly show non-additive gene action in the hybrids, and some of them showed over-dominance toward any one of the parental lines. The non-additive gene action could be driven by genetic diversity, allele polymorphism, events during gene regulation. The magnitude and the direction of the gene action of the candidate genes are very important to select the genes for drought tolerance. The magnitude of the gene expression and the expression pattern in the tissues will aid in selecting target candidate genes for drought tolerance breeding. The nature of the gene action and the direction of the expression pattern could play crucial roles in designing introgression and hybrid breeding programmes to breed drought tolerant maize hybrids.

## Author contributions

TN, HV, and MGM: conceived and designed the experiments; HV, TN, MGM, MS, BP, SJ, PD, AB, ARR, and RG: performed the experiments and analyzed the data; All authors contributed to manuscript preparation. All authors have read and approved the final manuscript.

### Conflict of interest statement

The authors declare that the research was conducted in the absence of any commercial or financial relationships that could be construed as a potential conflict of interest.
